# Investigation of the effects of white tea on liver fibrosis: An experimental animal model

**DOI:** 10.1002/fsn3.3980

**Published:** 2024-01-30

**Authors:** Hülya Kılıç Yılmaz, Merve Türker, Eda Yılmaz Kutlu, Tolga Mercantepe, Esra Pınarbaş, Levent Tümkaya, Mehtap Atak

**Affiliations:** ^1^ Department of Clinical Biochemistry, Faculty of Medicine Recep Tayyip Erdogan University Rize Turkey; ^2^ Biochemistry Laboratory Gumushane State Hospital Gumushane Turkey; ^3^ Department of Histology, Faculty of Medicine Recep Tayyip Erdogan University Rize Turkey

**Keywords:** antioxidants, hydroxyproline, liver fibrosis, white tea

## Abstract

Liver fibrosis is a common, progressive disease that affects millions of patients worldwide. In this study, it was aimed at investigating the effect of white tea on liver fibrosis in an in‐vivo environment by creating an experimental liver fibrosis model on rats. In this study, an experimental liver fibrosis model was created with carbon tetrachloride (CCl_4_) in Sprague–Dawley rats to investigate the effect of white tea on liver fibrosis. Rats are treated with CCl_4_ (1 mL/kg) to constitute the liver fibrosis model. White tea was given ad libitum with drinking water. As a result of the study, liver tissue hydroxyproline levels were found to be significantly lower (*p* = .001) in the white tea group. Histopathologically, it was found that the liver tissue histopathological damage score (LHDS) and fibrosis scoring were significantly lower (*p <* .001) in the white tea group. However, although it was not statistically significant in the group given white tea, compared with the fibrosis group, it was found that the malondialdehyde (MDA) level in the liver tissues was lower, the glutathione (GSH) level was higher, and the serum alanine aminotransferase (ALT) levels were lower. The study explained the effect of white tea on liver fibrosis and suggested that white tea might be beneficial in reducing the progression of liver fibrosis.

## Graphical abstract

Study Findings on White Tea and Liver Fibrosis. The **in vivo study** suggests that white tea may play a role in **slowing down** the progression of liver fibrosis in rats. Key observations include: **Significantly lower hydroxyproline levels** in the liver, which is a marker of fibrosis. **Decreased liver tissue damage**, indicating protective effects. **Reduced fibrosis levels** in the group that received white tea. **Improved antioxidant levels**, which may contribute to liver health. **Enhanced liver function**, showcasing potential health benefits.

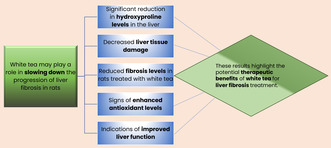

## 1 INTRODUCTION

Hepatic fibrosis is a wound healing response in chronic liver pathologies that is characterized by an excess of extracellular matrix production in the liver. If its progression cannot be prevented, it results in cirrhosis, which causes deterioration in the liver parenchyma and vascular structure. In addition to the liver failure it causes, cirrhosis also has the potential to turn into hepatocellular carcinoma. Therefore, the treatment of hepatic fibrosis is of utmost importance. Currently, there is no effective treatment for hepatic fibrosis (Asrani et al., 2019).

Living organisms are exposed to free radicals due to processes such as cellular metabolism, immune processes, and xenobiotic metabolism. Cells have antioxidant mechanisms, including molecules and enzymes, that can counter the harmful effects of free radicals. Oxidative stress, on the other hand, refers to the situation in which the cellular pro‐oxidant/antioxidant redox balance changes in favor of the pro‐oxidant state, leading to cellular damage. The association of many chronic liver diseases with oxidative stress has been extensively studied. The presence of oxidative stress has been demonstrated in almost all clinical and experimental settings of chronic liver disease, including chronic hepatitis C infection, alcoholic liver disease, hemochromatosis, and Wilson's disease. In addition, oxidative stress exerts a fibrogenic effect by affecting the cells responsible for the initiation and progression of hepatic fibrosis (Sanchez‐Valle et al., 2012). Therefore, the fact that oxidative stress plays a role in the formation of liver damage through the production of reactive oxygen species (ROS) and in the initiation of hepatic fibrogenesis is a hope for stopping hepatic fibrosis with antioxidant treatments.

The healing properties of plants were discovered in very ancient times, and they were used as medicines for the treatment of diseases. With great advances in medicinal chemistry in the last century, research continues to advance with synthetic drugs as well as natural plants. Natural products remain the focus of research as pioneering molecules (Heinrich et al., 2012).

The Black Sea region is the region where various types of tea are produced in Turkey, especially black tea. Although black tea production has been carried out in the Black Sea region for more than a hundred years, the history of white tea production is quite new. Scientific studies on white tea have shown that it has anticancer, antiobesity, antidiabetic, antiaterogenic, antimicrobial, antiviral, and neuroprotective properties, and these effects have been associated with the powerful antioxidant and anti‐inflammatory activity of white tea. White tea, named after the white plumes that cover the tea buds, was first discovered in the Tang Dynasty in China in the 6th century and became the interest of the dynasties (Ho et al., 2009). White tea leaves, which are less processed than other teas, are collected before they are fully opened and produced by withering and drying. While the best‐quality white tea is produced in the spring, it can also be produced in the summer and autumn (Wong et al., 2022). The important phenolic compounds found in tea leaves are catechins and their derivatives, which constitute 30% of the dry weight. Epigallocatechin gallate (EGCG), epigallocatechin (EGC), epicatechin (EC), and epicatechin gallate (ECG) are the main catechins found in white tea. EGCG has been identified as the major polyphenol (19.63%) in both white and green tea (Sanlier et al., 2019).

Catechins consist of three hydrocarbon rings and are structurally divided into estrocatechins (EGCG, ECG) and non‐ester catechins (EGC, EC). According to oxidation resistance, catechins can be listed as follows: EGCG > EGC > ECG > Gallic acid (GA) (Ravindranath et al., 2009). The antioxidant properties of catechins are determined by the position and number of hydroxyl groups in their structures. Catechins are strong hydrogen donors in the B and C rings. The 2,3‐double bond and the unsaturated 4‐oxo group in ring C enable electron delocalization of the ortho‐dihydroxy catechol in ring B. The conditions of the reaction environment and initial conditions are also responsible for the antioxidant activity of these compounds (LIczbiński & Bukowska, 2022).

Hepatic fibrosis is a disorganized healing process that occurs as a result of hepatic stellate cell activation. Hepatic stellate cell activation occurs as a result of the response of hepatocytes to recurrent inflammation. Proinflammatory compounds originating from hepatocytes accelerate the process by increasing oxidative stress. It is common to use antioxidants to reduce the occurrence and effects of oxidative stress. Delphinidine, a cyanidin derivative found in tea in the literature, through star cell inactivation and downregulation of TNF‐α and TGF‐β expression (Domitrović & Jakovac, 2010), chlorogenic acid through inhibition of the TLR‐4 signaling pathway (Shi et al., 2013), inhibition of gallic acid star cell activation and proliferation, inhibition of inflammatory enzyme synthesis and proliferation of inflammatory cytokines, induction of apoptosis and proinflammatory cytosine (El‐Lakkany et al., 2019). There are publications in which EGCG shows antifibrotic effects through inhibition of cell activation, reduction of oxidative stress, and suppression of the proinflammatory response (Tipoe et al., 2010). Another study has shown that white tea and EGCG are protective against liver dysfunction caused by oxidative stress. (Rangi et al., 2018). It is suggested that oxidative stress caused by increased production of reactive oxygen species and lipid peroxides is directly related to the proliferation and activation of hepatic star cells or through paracrine stimulation of neighboring cells, including injured hepatocytes (Svegliati Baroni et al., 1998). It has also been shown that oxidative stress modulates collagen gene expression (Parola et al., 1993). Therefore, various studies have focused on the pathogenetic significance of oxidative stress in liver injury and the therapeutic intervention of this process with antioxidant and metabolic scavengers. Green tea administration resulted in decreased lipid peroxide in HSC‐T6 cells and DMN‐treated fibrotic livers. A study has also shown that a single dose of EGCG, one of the polyphenolic compounds in tea products, improves liver damage in rats induced by the administration of CCL4 through the inhibition of lipid peroxidation (Chen et al., 2004). In the analyses made on black, green, and white tea grown in the Black Sea region, white tea has the richest polyphenolic content.

In this study, it was aimed at investigating the effect of white tea on liver fibrosis in an in‐vivo environment by creating an experimental liver fibrosis model on rats.

## 2 MATERIALS AND METHODS

### 2.1 Chemicals

The list of chemicals and devices used in the study is given in Appendix S1.

### 2.2 Animal experiments

In the study, male rats from the Sprague–Dawley breed weighing 150–200 grams, which are 6–8 weeks old, were used. The animals were obtained from the Experimental Animals Unit of Recep Tayyip Erdoğan University and housed in the same place in 12 h light and dark cycles, at 55%–60% humidity and at room temperature of 22 ± 2°C, in the environment of the experimental animal unit. Feed, a standard laboratory rodent diet, and water were given ad libitum. The approval of Recep Tayyip Erdoğan University's Animal Experiments Local Ethics Committee was obtained for the study. The research groups were randomly divided into 3 groups with 9 animals each: 1st Group: Control group; 2nd group: Fibrosis group; and 3rd group: Fibrosis and white tea group. Procedures applied to groups: During the 4‐week period, no additional procedures were applied to the control group other than routine care and nutrition. Fibrosis was induced with carbon tetrachloride (CCl4). CCl_4_ was administered to the fibrosis group 3 days a week by intraperitoneal injection at a dose of 1 mL/kg, inside olive oil with a dissolving rate of 50% (v/v). White tea was purchased from ÇAYKUR (Turkish Tea Institution). In addition to the procedures applied to the fibrosis group, white tea was prepared every day and given as drinking water and ad libitum to the white tea group. White tea is prepared daily with boiled water at 100°C, adding white tea leaves in the amount of 5 g per liter, and left to brew for 10 min. At the end of 4 weeks, the rats were sacrificed after a 12 h fasting period and anesthetized with ketamine. After blood was taken from the aorta into biochemistry tubes that did not contain anticoagulants, the livers of the rats were removed, and the appropriate amount of liver tissue was frozen in ice for biochemical analysis and placed in 10% formalin for histopathological analysis. Blood samples for serum were centrifuged at 3000 rpm for 10 min after coagulation was completed. After centrifugation, the serums were separated and aliquoted. The serum and liver tissues that will be used for biochemical analysis were stored at −80°C until the day of analysis.

### 2.3 Biochemical analysis

The research on the presence of malondialdehyde (MDA), glutathione (GSH), and hydroxyproline in liver tissues was performed. Preparation of tissue homogenate for the determination of MDA and GSH content: Liver tissues were weighed in eppendorfs at 100 mg. Then, after adding 1 mL of homogenization buffer to them, they were broken down in the homogenizer. After the resulting homogenate was centrifuged at 4°C at 800 *g* for 10 min, the supernatant portion was separated for use in the experiment. Determination of the presence of MDA: The determination of the presence of MDA was done via colorimetric spectrophotometry by modifying the method applied by Ohkawa et al. (Ohkawa et al., 1979). MDA solutions as standard substances: Solutions were prepared from a 200 nmol/mL stock solution with a serial dilution at concentrations of 5–2.5–1.25–0.625–0.3125 nmol/mL. 200 μL was pipetted from the standards and samples appropriate to Ependorphs. An equal volume of distilled water was pipetted for the blind. Then, 50 μL SDS, 375 μL 20% acetic acid, and 375 μL thiobarbituric acid (TBA) solutions were added to them and left to incubate for 1 hour in a hot water bath. The mixtures obtained after incubation were centrifuged at 750 *g* for 10 minutes. After the ependorphs cooled down, the supernatants of the mixtures were pipetted to the microplate wells in 3 repetitions for each, and their absorbance was measured with a spectrophotometer at a wavelength of 532 nm. The amount of MDA was determined as nmol/g of tissue.

Determination of the presence of GSH: The presence of GSH was determined by colorimetric spectrophotometry according to the method applied by Ellman (Ellman, 1959). Glutathione solutions were prepared as a standard substance at concentrations of 1–0.5–0.25–0.125–0.0625 nmol/mL by serial dilution from a 1 mM stock solution. 41.7 μL of the appropriate concentration of sample and glutathione solutions were pipetted on the microplate for 3 repetitions for each solution. An equal volume of distilled water was pipetted for the blind. Then, after adding 41.7 μL of Ellman reagent and 116 μL of buffer solution, the absorbance of the mixtures was measured by a spectrophotometer at a wavelength of 412 nm. The amount of GSH was determined as nmol/g of tissue.

Determination of the presence of hydroxyproline: The presence of hydroxyproline was determined by modifying the studies previously conducted by Wessner and See Wong (Oh et al., 2009; Woessner, 1961). Liver tissues were weighed to be 15 mg in ependorphs. Then, after 150 μL of distilled water was added to it, it was broken down in a homogenizer. 100 μL of the obtained homogenate was taken and transferred to glass bottles. After adding 100 μL of 10 N HCl to them, the lids were tightly shut, and they were hydrolyzed for 3 h in the drying oven at 120°C. Then, the obtained hydrolysate was centrifuged at 10000 *g* for 3 min, and 100 μL was taken from the supernatant portion, and 900 μL of acetate‐citrate buffer was added to it. After the pH of the resulting mixture was adjusted to 7, it was used as a sample in the experiment. Hydroxyproline solutions were prepared as a standard substance at concentrations of 100–80–40–20–10 μg/mL by serial dilution from a stock solution of 200 mg/mL. 40 μL of the standard solutions of the appropriate concentration were pipetted to the microplate for 3 repetitions for each solution. The samples were mixed with standard hydroxyproline at a concentration of 40 μg/mL in a ratio of 1:1 (v/v) and pipetted 40 μL to the microplate in 3 repetitions for each of the mixtures obtained. An equal volume of distilled water was pipetted for the blind. Then, 5 μL of chloramine‐T was added to each well and left to incubate in the dark at room temperature for 15 min. After 150 μL of p‐dimethylaminobenzaldehyde (DMAB) solution was added to each well after incubation, and the microplate was left to incubate for 35 min at 65°C, the absorbance of the mixtures was measured with a spectrophotometer at a wavelength of 550 nm. The amount of hydroxyproline was determined as mg/g of tissue.

Measurement of serum alanine aminotransferase (ALT) levels: Rat serums were studied after daily routine internal quality control on the Beckman Coulter AU5800 autoanalyzer, and their ALT levels were measured spectrophotometrically.

### 2.4 Histopathological assessment

To fix the fat tissues removed from the rats, they were fixed in a 10% neutral formalin (Sigma‐Aldrich) solution for 24 h, and then the tissue samples were placed in tissue tracking cassettes and placed in a tissue tracking device ThermoScientific™ Citadel 2000 During the procedures on the device, the tissue was dehydrated by keeping it in a series of 50% (2 times), 60%, 70%, 80%, 96%, and 100% (2 times) ethyl alcohol (Merck GmbH). Afterwards, the tissues were clarified with the xylol (Merck GmbH) series, and then they were blocked using tissue embedding cassettes using metal base molds from the paraffin blocking device (Leica EG 1150 H). Sections of 4–5 μm thickness were taken from the obtained paraffin blocks using a Rotary microtome (Leica RM2255), and staining was carried out on a staining device (Leica ST5020) with the Masson‐Goldner trichrome staining kit (Merck GmbH).

#### 2.4.1 Masson‐goldner trichrome staining method

1. Sections were subjected to deparaffinization and dehydration processes.
2. The staining process was started with a hematoxylin solution.
3. Sections were washed with tap water.
4. After exposure to a 1% acetic acid, it was kept in a azophloxine solution.
5. After exposure to a 1% acetic acid solution, it was kept in a tungstophosphoric acid orange G solution.
6. After exposure to a 1% acetic acid solution, it was kept in a light green SF solution and then kept in a 1% acetic acid solution again.
7. Dehydration was achieved by passing through the 70%, 96%, and 100% (2 times) ethanol series.
8. Clarification was carried out with xylene.
9. A coverslip was applied to the sections with entellan.

#### 2.4.2 Semiquantitative analyses

To examine the sections stained with Goldner's Masson trichrome histopathologically, the fibrosis damage score (FS) was calculated as shown in Table 1. Semi‐quantitative analyses were performed by two different histopathologists who were blinded to the study groups in 20 different areas randomly selected from each subject's section.

**TABLE 1:** Tissue MDA, GSH, and Hydroxyproline Levels, and Serum ALT Levels.

Group	MDA (nmol/g of tissue)	GSH (nmol/g of tissue)	Hydroxyproline (mg/g of tissue)	ALT (U/L)
Control	19.34 ± 2.49	19.90 ± 1.31	3.38 ± 0.57	47 ± 6
Fibrosis	28.35 ± 5.88^a^	15.80 ± 1.43^a^	6.77 ± 0.97^a^	750 ± 344^a^
Fibrosis & White Tea	26.14 ± 2.62^b^	16.02 ± 2.43^b^	5.08 ± 0.38^a,b^	722 ± 396^a^

*Note*: Table 1, Figure 1a–d. MDA: ^a^ *p* = .005 (compared to the control group), ^b^ *p* = .001 (compared to the control group). ^a^GSH: ^a^ *p* = .01 (compared to the control group), ^b^ *p* = .002 (compared to the control group). Hydroxyproline ^a^ *p <* .001 (compared to the control group), ^b^ *p* = .001 (compared to the fibrosis group). ALT: ^a^ *p <* .001 (compared to the control group).

#### 2.4.3 Quantitative analysis

Fat tissue surface areas in sections stained with Goldner's Masson trichrome. The Olympus DP2 (Olympus Corp.) program was used. Measurements were made in six randomly selected different areas in each preparation with the polyglonal probe of this program.

### 2.5 Statistical analysis

The results of biochemical analyses were given as the mean (X) and standard deviation (SD). The results were evaluated by looking at 5 parameters: whether the obtained data were suitable for the normal distribution, the histogram chart of the groups, whether the coefficient of variation was less than 30%, whether the standard error in skewness and kurtosis values times two was greater than the absolute value of skewness and kurtosis coefficients, the distribution of the data in the Q‐Q chart, and the p value in the Shapiro–Wilk test. Accordingly, intergroup comparisons of the data appropriate to the normal distribution were performed via oneway analysis of variance (oneway ANOVA). A post‐hoc Bonferroni correction was made. The value of *p <* .05 was considered statistically significant. The nonparametric data obtained as a result of semi‐quantitative histopathological analyses were calculated as the median and 25% and 75% interquartile slices, respectively. The differences between the groups were analyzed by applying Kruskall–Wallis and then a complete T test. Deciphering the differences between the groups was carried out using the Kruskal–Wallis test. *p <* .05 was considered statistically significant.

## 3 RESULTS

### 3.1 MDA and GSH levels in liver tissues

**FIGURE 1:**
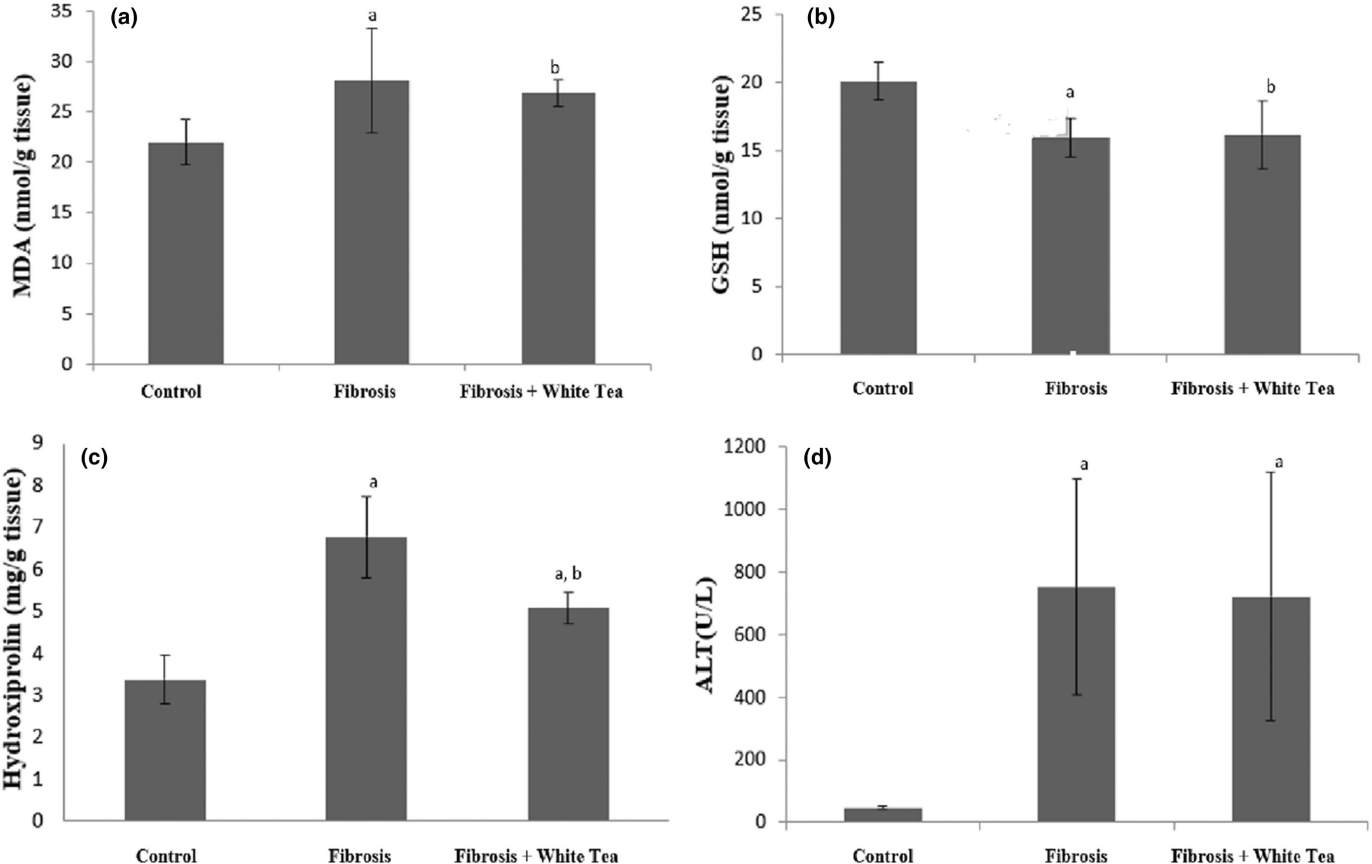
(a): MDA ^a^ *p* = .005 (compared to the control group), ^b^ *p* = .001 (compared to the control group). (b): GSH: ^a^ *p* = .01 (compared to the control group), ^b^ *p* = .002 (compared to the control group). (c): Hydroxyproline ^a^ *p <* .001 (compared to the control group), ^b^ *p* = .001 (compared to the fibrosis group). (d): ALT: ^a^ *p <* .001 (compared to the control group).

When the MDA and GSH levels of the groups were examined, it was revealed that there was a significant difference in the level of *p* = .005 for MDA and *p* = .01 for GSH in the fibrosis group compared to the control group. According to the control group, there is a significant difference in the level of *p* = .001 for MDA and *p* = .002 for GSH in the fibrosis and white tea groups. Although there was no statistically significant difference between the fibrosis and fibrosis and white tea groups for MDA and GSH (*p >* .05), MDA levels were lower and GSH levels were higher in the fibrosis and white tea group compared to the fibrosis group (Table 1, Figure 1a, and Figure 1b).

### 3.2 Hydroxyproline levels in liver tissues

When the hydroxyproline levels of the groups were examined, it was found that there was a significant difference in the level of *p <* .001 in the fibrosis group compared to the control group. There was a significant difference in the level of *p <* .001 in the fibrosis+white tea group compared to the control group. Between the fibrosis and fibrosis+white tea groups, there was a significant difference at the level of *p* = .001 (Table 1, Figure 1c).

### 3.3 Serum (alanine aminotransferase) ALT levels

There was a significant difference in the ALT levels at *p <* .001 in the fibrosis group compared to the control group. There was a significant difference in the level of *p <* .001 in the fibrosis+white tea group compared to the control group. Although there was no statistically significant difference between the fibrosis and fibrosis+white tea groups (*p >* .05), the ALT levels were found to be lower in the fibrosis+white tea group compared to the fibrosis group (Table 1, Figures 1d and 3d).

### 3.4 Findings of histopathological analysis

**FIGURE 2:**
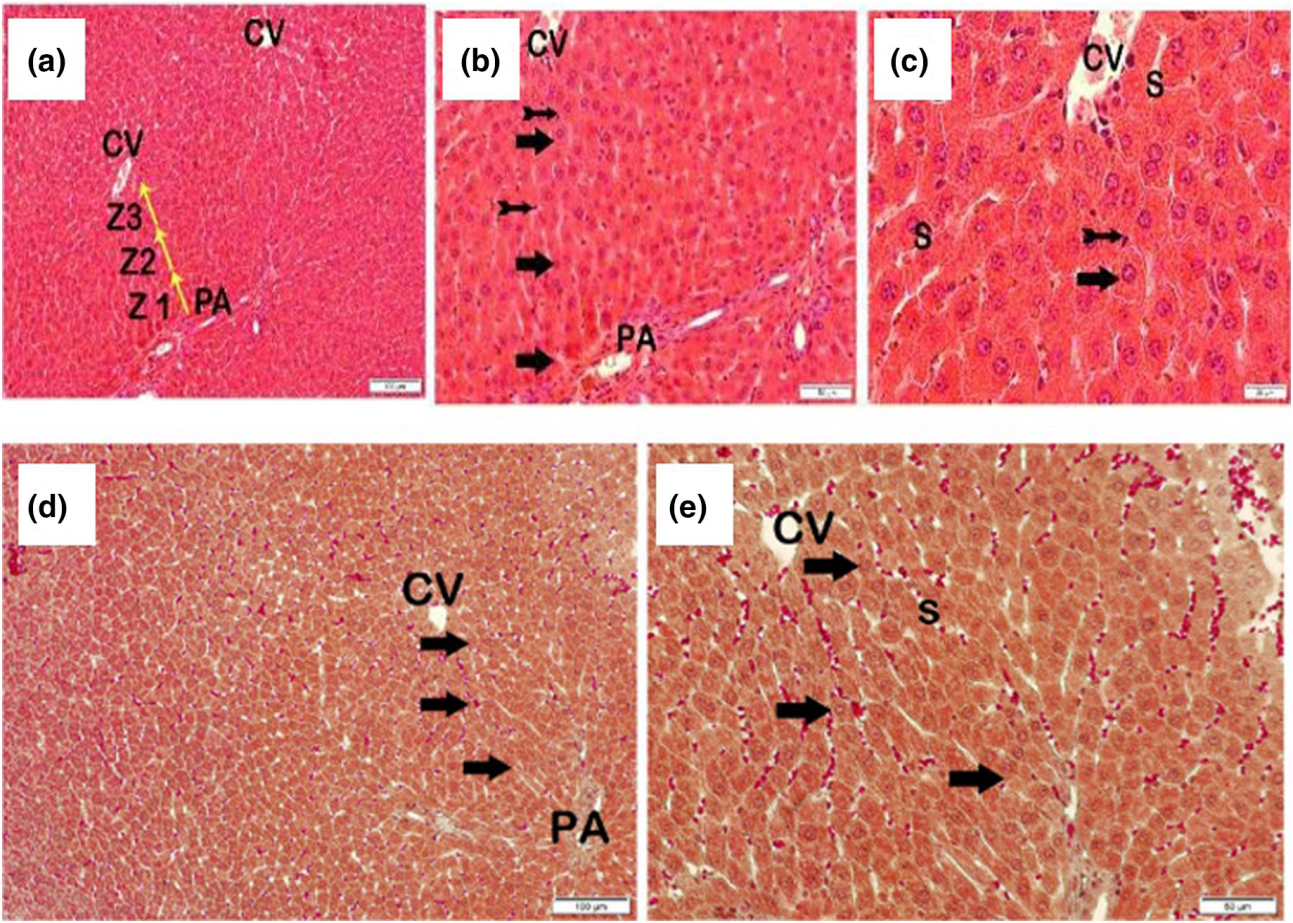
Representative light microscope images of liver tissue sections belonging to the control group stained with H&E a(x10)–b(x20)–c(x40) (−, Tailed arrow; Arrow, NORMAL hepatocyte; CV, central vein; Kupffer cells, Z1; Zones 1, Z2; Zone 2, Z3; Zone 3; PA, The Portal area; S, Sinusoid). Representative light microscopic images of liver tissue sections belonging to the control group stained with Masson Trichrome d (x10)–e (x20) (arrow, Normal hepatocyte; CV, Central vein; PA, Portal area; S, Sinusoid).

When the sections stained with H&E and Masson Trichrome belonging to the control group were examined under light microscopy, it was observed that the remark cords in liver zones 1, 2 and 3 consisted of normal hepatocytes (LHDS: 0 (0–1), fibrosis score: 0(0–0)) (Figure 2).

**FIGURE 3:**
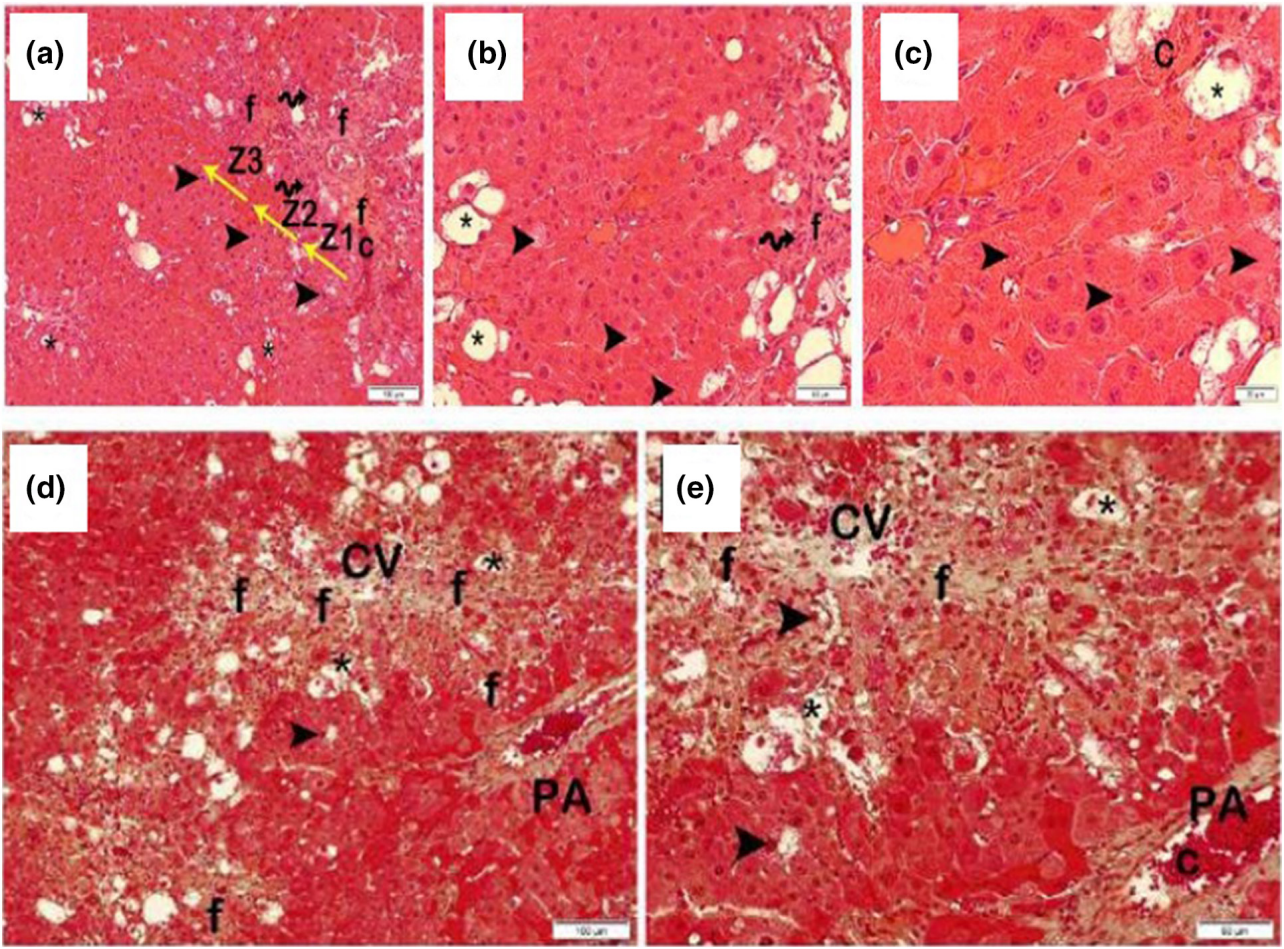
Representative light microscopic images of liver tissue sections belonging to the fibrosis group stained with H&E, a(x10)–b(x20)–c(x40) (Arrowhead, Intralobular piece (paecemeal) necrosis; C, Vascular congestion; f, Fibrosis; Spiral Arrow, Infiltrative areas; Star, fatty degeneration; Z1, Zone 1; Z2, Zone 2; Z3, Zone 3). Representative light microscopic images of sections of liver tissue belonging to the fibrosis group stained with Masson Trichrome, d(x10)–e(x20) (Arrowhead, Intralobular piece (paecemeal) necrosis; C, Vascular congestion; CV, central vein; f, Fibrosis; PA, The Portal area; star, fatty degeneration).

When the sections of liver tissue were stained with H&E and Masson Trichrome belonging to the fibrosis group, in the remark cords, intralobular fragmentary (paecemeal) necrosis formed by common necrotic hepatocytes, perilobular bridge necrosis (bridging), and multilobular necrosis were present. In addition, it was observed that a large number of hepatocytes with a rich fat vacuole content came together and caused fatty degenerations in the liver. In addition, fibrosis and infiltrative areas were observed in the portal areas (LHDS: 11 (El‐Lakkany et al., 2019; Shi et al., 2013; Tipoe et al., 2010), fibrosis score: 2 (Sanchez‐Valle et al., 2012)) (Figure 3).

**FIGURE 4:**
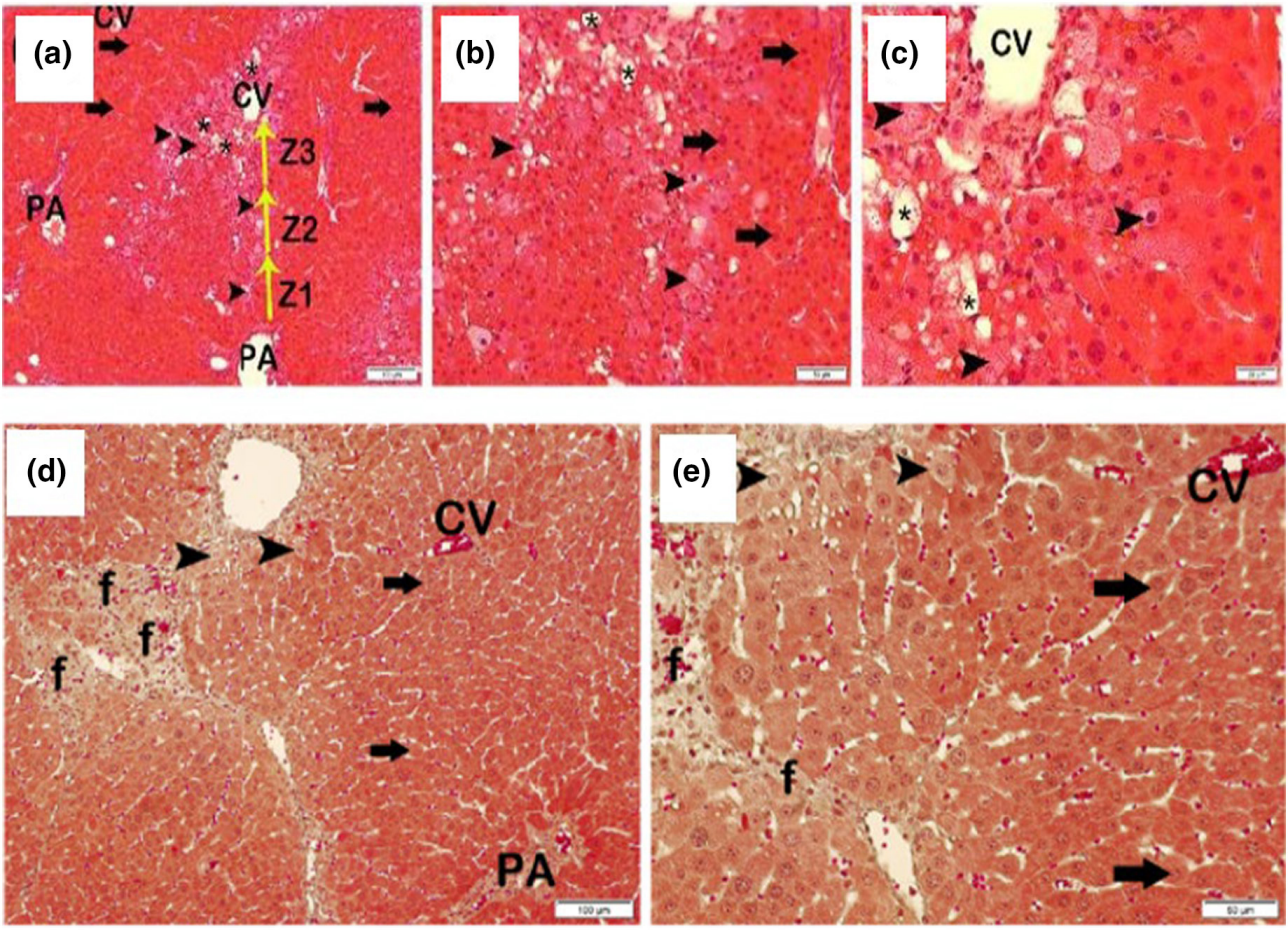
Representative light microscopic images of liver tissue sections belonging to the H&E stained fibrosis + white tea group, a(x10)–b(x20)–c(x40) (Arrow, Normal hepatocyte; Arrowhead, Intralobular piece (paecemeal) necrosis; CV, central vein; PA, The Portal area; Star, fatty degeneration; Z1, Zone 1; Z2, Zone 2; Z3, Zone 3). Representative light microscopic images of sections of liver tissue belonging to the group of white tea + fibrosis stained with Masson Trichrome, d(x10)–e(x20) (Arrow, Normal hepatocyte; Arrowhead, Intralobular piece (paecemeal) necrosis; CV, central vein; f, Fibrosis; PA, The Portal area.).

When the sections of liver tissue were stained with H&E and Masson Trichrome belonging to the fibrosis + white tea group, the number of necrotic hepatocytes in the remark cords was reduced, and intralobular focal and piecemeal necrosis was detected in some places. However, it was observed that perilobular bridges (bridging) and multilobular necrosis decreased. In addition, decreased fatty degenerations, fibrosis, and infiltrative areas were observed (LHDS: 7 (LIczbiński & Bukowska, 2022; Ravindranath et al., 2009; Sanlier et al., 2019), fibrosis score: 1 (Asrani et al., 2019; Sanchez‐Valle et al., 2012)) (Figure 4). The results of the semi‐quantitative analysis of the groups are shown in Table 2.

**TABLE 2:** Semi‐quantitative analysis results (median (25% and 75% interquartile range)).

Group	Fatty degeneration of the liver	Portal inflammation	Fibrosis	Intralobular degeneration and focal necrosis	Necrosis of the periportal bridge (bridging)	LHDS
Control	0 (0–0)	0 (0–0)	0 (0–0)	0 (0–0)	0 (0–0)	0 (0–1)
Fibrosis	3 (2‐3)^a^	1 (1‐1)^a^	2 (2‐2)^a^	3 (3‐3)^a^	3 (2‐3)^a^	11 (10–12)^a^
Fibrosis& White tea	2 (1‐2)^a^ ^,^ ^b^	0 (0‐1)^b^	1 (1‐2)^a^ ^,^ ^b^	2 (2‐2)^a^ ^,^ ^b^	2 (2‐2)^a^ ^,^ ^c^	7 (6‐8)^a^ ^,^ ^b^

^a^ *p <* .001 (compared to the control group).

^b^ *p <* .001 (compared to the fibrosis group).

^c^ *p* = .002 (compared to the fibrosis group).

## 4 DISCUSSION

Hepatic fibrosis, a health problem affecting an estimated 100 million people worldwide, is the wound healing response of the liver against repetitive damage caused by viral infections (such as HBV and HCV), alcohol use, autoimmune and metabolic diseases, and is associated with the production of an excess of extracellular matrix that disrupts the liver structure (Li et al., 2011). Since it can cause devastating consequences such as cirrhosis and hepatocellular carcinoma, which cause liver failure if their progression cannot be prevented, the search for an effective treatment that will slow down hepatic fibrosis is still ongoing. Many factors that cause chronic liver damage are associated with oxidative stress. Oxidative stress causes the activation of hepatic stellate cells, which are the main source of extracellular matrix production in fibrogenesis (Baroni et al., 1998). Therefore, the fact that the pathogenesis of hepatic fibrosis involves the processes associated with oxidative stress makes the application of antioxidants an important opportunity for treatment.

Many beneficial plants that have been used as medicinal drugs since ancient times are still being investigated today in terms of their therapeutic properties, mechanisms of action, and effective and toxic doses for the treatment of many diseases. In particular, the fact that some plants are easily accessible, low‐cost, have fewer side effects, and have high antioxidant properties compared to chemical drugs leads researchers to plant‐based medicine production. We see that there are many studies in the literature on various pathologies of the liver using natural products with high antioxidant properties, and positive results have been achieved (Simón et al., 2020). In this study, we investigated the effects of white tea, which is a powerful antioxidant, on hepatic fibrosis.

As the main non‐enzymatic regulator of intracellular redox balance, GSH is used as a sensitive marker of oxidative stress. GSH participates in redox reactions by oxidation of the active thiol group and turns into its oxidized form, glutathione disulfide (GSSH). As a result of cell damage caused by free radicals, the level of GSH decreases because of consumption. MDA, another marker used to assess oxidative stress, is one of the byproducts caused by the oxidation of lipids containing polyunsaturated fatty acids, which is caused by free radicals, and an increased MDA level indicates an excess of oxidative damage (Arauz et al., 2016). In our study, we found that white tea increased GSH levels and decreased MDA levels in CCl4‐induced damaged liver cells, although the difference was not statistically significant. Similarly, we found that serum ALT levels were lower in the group given white tea, although it was not statistically significant. In the literature, there are studies indicating that white tea is protective against CCl4‐induced liver damage. In a study by Wang et al., rats were given low and high doses of white tea for 14 days, and on day 14, CCl4 was given intraperitoneally to induce liver damage. When the experiment was terminated after 24 h and the blood and tissues of the animals were examined, it was found that serum ALT, AST, and tissue MDA levels were lower and tissue GSH‐Px (glutathione peroxidase) activity was higher in rats that were given white tea. In addition, it was found that the healing effect was greater in the group given a high dose (200 mg/kg) of white tea (Wang et al., 2019). A similar study was conducted by Yi et al., and the hepatoprotective effect of white tea was associated with the polyphenols it contained, such as gallic acid, catechin, hyperoside, and sulfuretin (Yi et al., 2020). The reason why the antioxidant effect was not statistically significant in our study may be that the amount of white tea was insufficient in response to the CCl4 dose we gave.

There are also studies on the hepatoprotective effect of white tea using hepatotoxic substances other than CCl4. In an in vitro study on the effect of white tea and its active component EGCG (equivalent dose to the given white tea extract), it was found that the antioxidant potential of EGCG was higher compared to white tea, while in their in vivo study on the oxidative stress‐mediated hepatotoxicity of benzoapirene in rats, they found that both reduced the negative effects of benzoapirene and that there was no difference between each other in terms of effectiveness. This was explained by the fact that the polyphenols contained in tea have a synergistic effect (Neong et al., 2019). In a study conducted by Hamdy et al. on acrylamide hepatotoxicity in rats, the protective effect of white tea was demonstrated, attributing this effect to the fact that white tea directly inhibits the cytochrome P450 enzyme, which causes ROS production, glutathione metabolism, and free radical formation (Hamdy et al., 2020). In a study in which Zhou et al. investigated the effect of white tea polyphenols against alcoholic liver damage, they showed that white tea polyphenols slowed down pathological outcomes, attributing these effects to the powerful antioxidant properties possessed by gallic acid, catechins, and hyperosides contained in white tea (Zhou et al., 2019).

The main element of the extracellular matrix produced in the process of developing hepatic fibrosis is fibrillary collagen, especially type 1 and type 3 collagens, and about 100 out of every 1000 amino acids found in fibrillary collagen are hydroxyproline. Thus, hydroxyproline is a marker that provides biochemical detection of the extracellular matrix, and the measurement of the amount of hydroxyproline in tissues is used to determine the estimated amount of collagen present in the tissue (Brown et al., 2001). In our study, we measured the amount of hydroxyproline to estimate the degree of fibrosis in liver tissues. At the same time, a histological evaluation was performed. As a result of these evaluations, we found that the amount of hydroxyproline in the group given fibrosis + white tea was significantly lower than in the fibrosis group. Histological findings were also found to be in a similar direction. These findings suggest that white tea is effective in reducing the severity of hepatic fibrosis. Although there is no study in the literature that examines the effects of white tea on hepatic fibrosis, there are studies that examine the effects of other kinds of teas. For example, It was conducted a study on the effect of EGCG, also found in white tea, on CCl4‐induced hepatic fibrosis in rats, and CCl4 and EGCG were given intraperitoneally to rats for 8 weeks. As a result, profibrogenic factors were slowed down and the activation of hepatic stellate cells decreased in rats given EGCG (Tipoe et al., 2010). The exact mechanism of this protective effect of EGCG on CCl4‐induced liver damage and fibrosis has not been fully determined, but the protective effect has been attributed to the regulation of oxidative stress and inflammatory processes by EGCG (Brown et al., 2001). Similarly, a study by Wang et al. showed that catechins slowed down CCl4‐induced hepatic fibrosis (Wang et al., 2019). As seen in previous studies, ECGC from tea components has been focused on. In this study, an animal experiment was planned using all of the tea in the form of drinking water. In this way, the protective effect of white tea, which is a pleasant drink, has been studied in its entirety. The limitations of the study are that it cannot be determined at what level compounds of tea are in rat blood.

In conclusion, the findings obtained in this study using an experimental liver fibrosis model, especially tissue hydroxyproline and histopathological findings, suggest that white tea may slow down liver fibrosis. Further and more detailed research with different doses is needed to elucidate the mechanism of action.

## AUTHOR CONTRIBUTIONS

**Hülya Kılıç Yılmaz:** Project administration (equal); validation (equal); writing – original draft (equal); writing – review and editing (equal). **Merve Türker:** Investigation (equal); writing – review and editing (equal). **Eda Yılmaz Kutlu:** Investigation (supporting); visualization (supporting). **Tolga Mercantepe:** Investigation (supporting); visualization (supporting). **Esra Pınarbaş:** Investigation (supporting); visualization (supporting). **Levent Tümkaya:** Investigation (supporting); visualization (supporting). **Mehtap Atak:** Supervision (equal); validation (equal).

## CONFLICT OF INTEREST STATEMENT

The authors declare no competing interests.

## ETHICS STATEMENT

All of the protocols of the article were approved by the board at the Recep Tayyip Erdogan University Animal Ethical Committee, and written informed consent was obtained from all the participants.

## Supporting information


Appendix S1.

## Data Availability

The data that support the findings of this study are available on request from the corresponding author.
