# Supplementary material for: Investigation of the effects of white tea on liver fibrosis: An experimental animal model
Source: Food Sci Nutr. 2024 Jan 30;12(4):2998–3006. doi: 10.1002/fsn3.3980 (PMC11016422; doi:10.1002/fsn3.3980)
Supplement: Supplementary file 1 — Appendix S1. [file FSN3-12-2998-s001.pdf]

**SUPPLEMENT 1**

The chemicals and devices used in the study are listed below.

**I. Chemicals**

1. Sodium dihydrogen phosphate ($NaH_2PO_4$) (Sigma Aldrich)
2. Disodium hydrogen phosphate ($Na_2HPO_4$) (HIMEDIA)
3. Potassium chloride (KCl) (HIMEDIA)
4. Sodium dodecyl sulfate (SDS) (Sigma Aldrich)
5. Acetic acid (KİMETSAN)
6. Thiobarbituric acid (TBA) (MERCK)
7. 1,1,1,3,3 tetramethahydroxypropane (TEP) (Fluka)
8. DTNB (5,5'-dithiobis-(2-nitrobenzoic acid)) (Alfa Aeser)
9. L-Glutathione reduced (Sigma Aldrich)
10. DMAB (p-Dimethylaminobenzaldehyde) (Sigma Aldrich)
11. Sodium acetate ($NaCH_3COO^-$) (HIMEDIA)
12. Trisodium citrate ($Na_3C_6H_5O_7$) (HIMEDIA)
13. Citric acid ($C_6H_8O_7$) (Sigma Aldrich)
14. Perchloric acid (Carlo Erba)
15. N-Propanol (Carlo Erba)
16. Chloramine-T (Sigma Aldrich)
17. Hydroxyproline (Sigma Aldrich)

**II. Devices**

1. Precision scales (Precisa XB 220 A)
2. Automatic pipette (Brand, Socorex)
3. pH Meter (HANNA HI 2210)
4. Microplate reader (Thermo Scientific Multiskan GO)
5. Heated magnetic stirrer (Are Velp Scientifica)
6. Homogenizer (Tissue Lyser II QIAGEN)
7. Centrifuge (Thermo Scientific Multifuge 3SR+)
8. Water Bath (Memmert WNB 14)
9. Etuve (Memmert UF 55)
10. Heat Block (HLC)
11. Beckman Coulter AU5800 Autoanalyzer
